# Apocrine Breast Carcinoma with Thanatosomes (Hyaline Globules)

**DOI:** 10.3390/diagnostics15141768

**Published:** 2025-07-13

**Authors:** Mitsuhiro Tachibana, Masashi Nozawa, Tadahiro Isono, Kei Tsukamoto, Kazuyasu Kamimura

**Affiliations:** 1 Department of Diagnostic Pathology, Shimada General Medical Center, Shimada 427-8502, Shizuoka, Japan; 2 Department of Surgery, Shimada General Medical Center, Shimada 427-8502, Shizuoka, Japan; masashin@shimada-gmc.com (M.N.); isonotadahiro@gmail.com (T.I.);; 3 Department of Diagnostic Radiology, Shimada General Medical Center, Shimada 427-8502, Shizuoka, Japan

**Keywords:** breast, carcinoma with apocrine differentiation, apocrine carcinoma, cleaved caspase-3, apoptosis, hyaline globules, death bodies, thanatosomes

## Abstract

Thanatosomes (hyaline globules or death bodies) are histologically observed in various non-neoplastic and neoplastic conditions. Some of these globules are associated with apoptotic cell death. Only six documented cases of thanatosomes have been reported in breast tumors. In this rare case involving a 64-year-old Japanese woman diagnosed as having rectal cancer, preoperative computed tomography scanning revealed breast cancer in her right breast. Following a right total mastectomy, a tumor characterized as apocrine carcinoma (carcinoma with apocrine differentiation) containing thanatosomes was discovered. These globules are PAS positive and diastase resistant, exhibit deep fuchsinophilic staining with Masson’s trichrome, stain dark blue with PTAH, and are negative for mucin by Alcian blue. The tumor cells tested positive for the androgen receptor, FOXA1, and GCDFP15. Human epidermal growth factor type 2 (HER2)/*neu* score was 3+/positive, and the Ki-67 labeling index was 60%. Thus, the tumor represented high-grade, HER2-enriched apocrine carcinoma. Thanatosomes are immunoreactive to cleaved caspase-3 and are histological markers of high cell turnover and apoptotic cell death. Therefore, in this nonspecific microscopic neoplastic condition, they are typically linked to high-grade tumors, as this case showed. This report presents a rare case of apocrine breast cancer featuring a limited number of thanatosomes.

**Figure 1:**
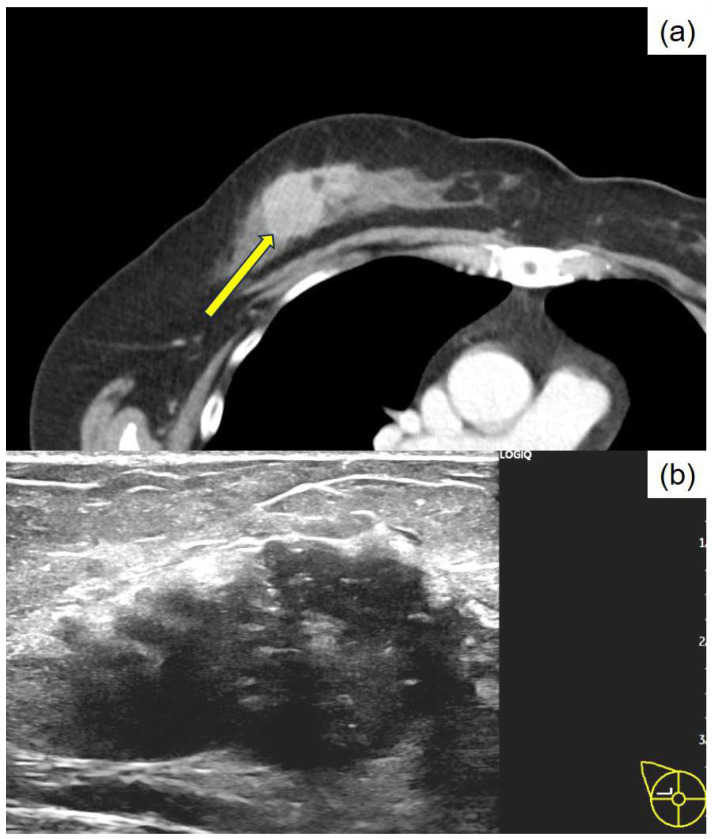
Initial clinical imaging findings show (**a**) a contrast-enhanced computed tomography image revealing an irregular mass in the right breast (arrow) and (**b**) a breast ultrasound depicting an irregular, indistinct mass in the lateral region of the right breast. The internal echo is heterogeneous and displays numerous microcalcifications. Thanatosomes, also known as death bodies or hyaline globules (HGs), are morphological entities that represent metabolic imbalances found in all cell types. HGs have been observed in various tumors and benign tissues [1,2], and some studies suggest a close relationship between HGs and apoptosis [1,2,3,4,5,6,7]. However, we previously reported that HGs developed independently of apoptosis in two cases of pancreatic intraductal papillary mucinous neoplasms [8]. The present report presents a rare case in which HGs were noted in the cytoplasm of tumor epithelial cells in breast apocrine carcinoma. Only six cases of breast tumors involving thanatosomes have been reported [3,4,5,6,7], and there are no reports of thanatosomes in carcinoma with apocrine differentiation. We present a case of apocrine carcinoma with thanatosomes and briefly review the relevant literature. A 64-year-old Japanese woman was diagnosed as having microcytic hypochromic anemia during a health checkup. She reported experiencing melena and visited Shimada General Medical Center in Shimada, Shizuoka, Japan. Her medical history includes hypertension, dyslipidemia, and chronic hepatitis B along with a smoking history of 20 cigarettes per day from the age of 20 to 44 years. Her mother had a history of uterine cancer. The patient’s measured tumor markers were CEA 3.3 ng/mL, CA19-9 85 U/mL, CA153 21.5 U/mL, and HER2 protein 16.2 ng/mL. A type 2 tumor was found in the upper rectum (Ra; rectum above the peritoneal reflection) with 3/4 circumferential involvement. A preoperative computed tomography examination for rectal cancer raised suspicion of right lateral breast cancer (Figure 1). A core needle biopsy confirmed apocrine carcinoma, which was classified as human epidermal growth factor receptor type 2 (HER2)-enriched type. According to Japanese guidelines, neoadjuvant chemotherapy is not actively recommended for R0 resectable rectal cancer [8], whereas for HER2-enriched subtype breast cancer, perioperative chemotherapy following the KYATHERINE trial protocol [9] is recommended. However, because of concerns about lumen obstruction of locally advanced rectal cancer during neoadjuvant chemotherapy (6 months) for breast cancer, priority was given to performing surgery on the same day. A right total mastectomy was performed. The tumor measured 39 × 25 × 24 mm and appeared as a solid mass infiltrating the adipose tissue (Figure 2). Histologically, it displayed characteristics of apocrine carcinoma, a type exhibiting apocrine differentiation, was classified as a HER2-enriched subtype, and had a high Ki-67 labeling index of 60% (Figure 3a and Figure 4). Additionally, several eosinophilic thanatosomes were noted (Figure 3b). The staining pattern of the thanatosomes corresponded with those of previous reports [1,5,7]. Specifically, these are PAS-positive and diastase-resistant globules stained deeply fuchsinophilic with Masson’s trichrome, appear dark blue with PTAH, and test negative for mucin by Alcian blue (Figure 5). We had planned to perform a sentinel lymph node biopsy because we initially clinically diagnosed no lymph node metastasis. Still, during the operation, the sentinel node could not be identified by dye or indocyanine green fluorescence, and although the Level-I lymph node showed no evidence of malignancy pathologically, there was a hard metastatic lymph node in the vicinity, so axillary Level-II lymph node dissection was performed. We did not administer neoadjuvant chemotherapy to the patient for both the rectal and breast cancers because both were considered to be amenable to postoperative adjuvant chemotherapy. We thus thought it better to operate on both cancers at the same time and then decide on a postoperative chemotherapy plan based on the pathological diagnosis results. Axillary lymph nodes showed breast cancer metastasis in 3 of 13 nodes. According to the UICC TNM Classification of Malignant Tumours, 8th edition, the pathological staging of the rectal cancer was pT3N1bM0, pStage IIIB, whereas that of the breast cancer was pT2N1aM0, pStage IIB. Her postoperative course was uneventful, and recovery proceeded well. We planned to start with 3–6 months of chemotherapy for the rectal cancer, followed by about a year of chemotherapy for the breast cancer, which, in detail, comprised four cycles of the CAPOX (capecitabine and oxaliplatin) regimen for pStage IIIb rectal cancer, and then four cycles of the AC (doxorubicin and cyclophosphamide) regimen, followed by four cycles of DHP (docetaxel, trastuzumab, and pertuzumab), and finally, by 14 cycles of HP (trastuzumab and pertuzumab) for the pStage IIB breast cancer. This is a rare case of apocrine breast cancer with thanatosomes (HGs). These globules are associated with apoptotic cell death, as indicated by their immunoreactivity to cleaved caspase-3 (Figure 5e and Figure 6) [10]. These nonspecific microscopic findings serve as histological markers of high cell turnover and apoptotic cell death; thus, in neoplastic conditions, they are typically linked to high-grade tumors [3,5]. This report is the first, to our knowledge, to show thanatosomes in apocrine breast carcinoma [3,4,5,6,7] and that these thanatosomes in breast cancer are immunoreactive for cleaved caspase-3. The thanatosomes in this patient also exhibited immunoreactivity for ubiquitin (Figure 5f). Dikov et al. proposed that some HGs might arise from inhibition of the cytoplasmic alternative ubiquitin-proteasome system [2]. This study clearly demonstrates that thanatosomes in breast cancers are caused by apoptosis, as evidenced by positive staining for cleaved caspase-3. Breast cancers with thanatosomes, as seen in the present case and in Panicker et al.’s work [5], are often high-grade, suggesting that the histological identification of thanatosomes may serve as a potential indicator of poor prognosis. By comparing with previously reported cases and accumulating more data, it may become possible to establish thanatosomes as a histological prognostic marker in breast cancer. A major limitation of this report is that it is a single case report from one institution in Japan. Further clinicopathological analyses, including multicenter studies, will be necessary to definitively determine the cause and pathophysiology of this condition. Therefore, reports from other countries, cultures, and hospitals are eagerly anticipated. In conclusion, thanatosomes are immunoreactive to cleaved caspase-3 and ubiquitin, indicating their connection to apoptosis. Thanatosomes are related to a high turnover of cells [6]. Previous studies have shown thanatosomes to be present in high-grade malignant breast tumors [3,5]. The present report is the first to show thanatosomes as a significant morphological feature in breast apocrine carcinoma.

**Figure 2:**
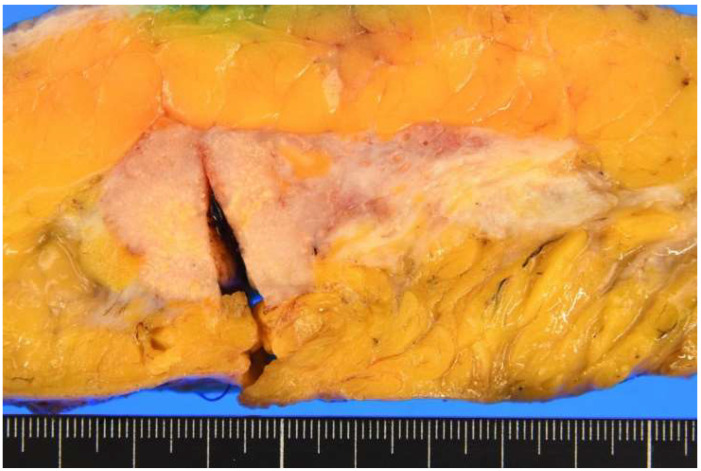
Macroscopic findings of the resected specimen from the right total mastectomy. The tumor measures 39 × 25 × 24 mm in diameter and resembles a lobulated, solid mass with a milky white and focal yellowish appearance, accompanied by fat invasion.

**Figure 3:**
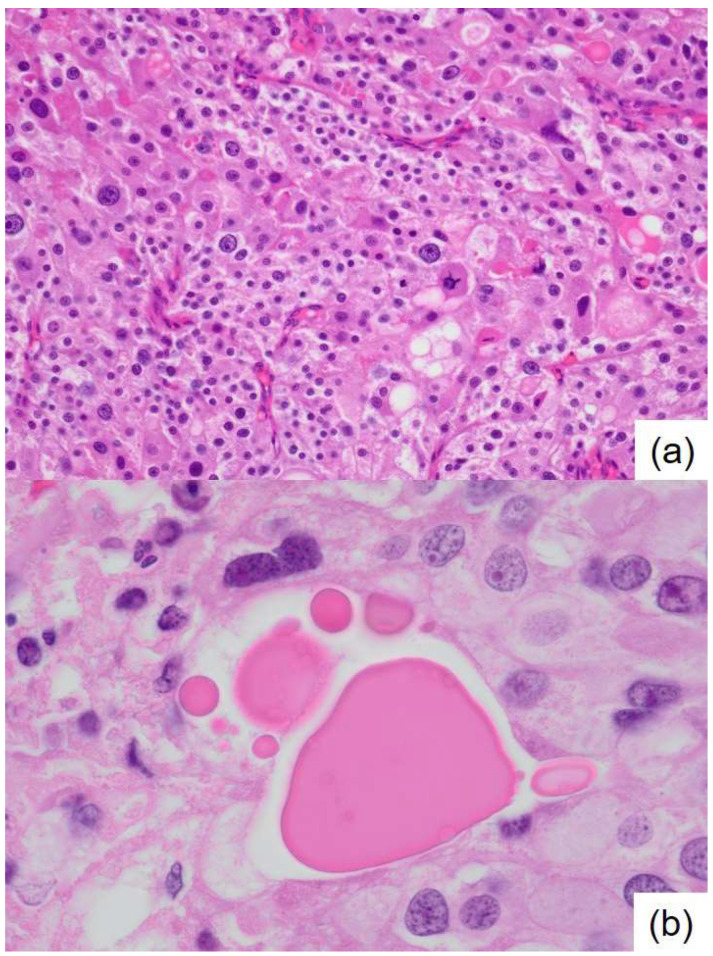
Histopathology of the resected breast specimen stained with hematoxylin and eosin. (**a**) The tumor forms sheet-like solid nests, shows proliferation, and is accompanied by abundant capillaries. The cytoplasm appears eosinophilic and granular; the nuclei are round to oval and demonstrate notable nuclear pleomorphism. Occasional mitotic figures are observed, ×200; (**b**) Various sizes of eosinophilic and uniform thanatosomes are intermittently noted within the breast cancer tissue, ×1000.

**Figure 4:**
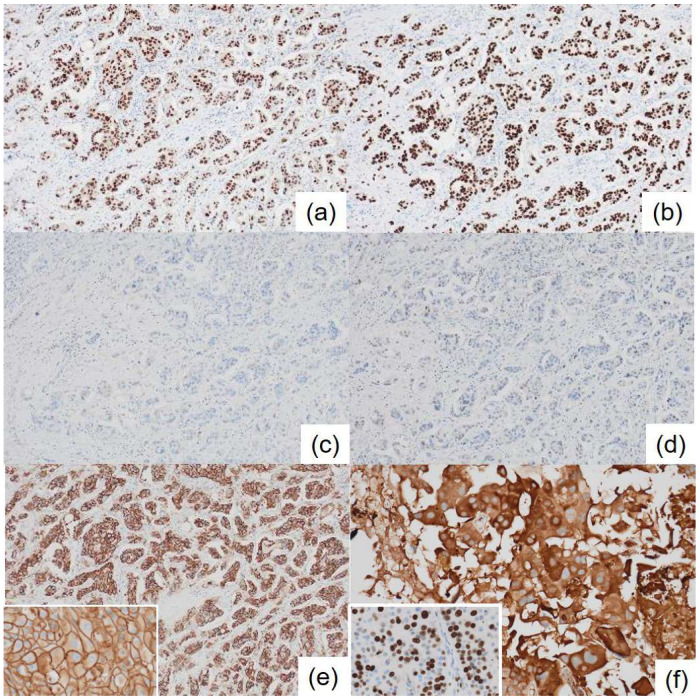
Immunohistochemical staining of the breast cancer. (**a**) Androgen receptor, ×200; (**b**) FOXA1, ×200; (**c**) estrogen receptor, ×200; (**d**) progesterone receptor, ×200; (**e**) HER2/*neu*, ×200 (inset: enlarged image; ×400); and (**f**) GCDFP15, ×400 (inset: Ki-67; ×400).

**Figure 5:**
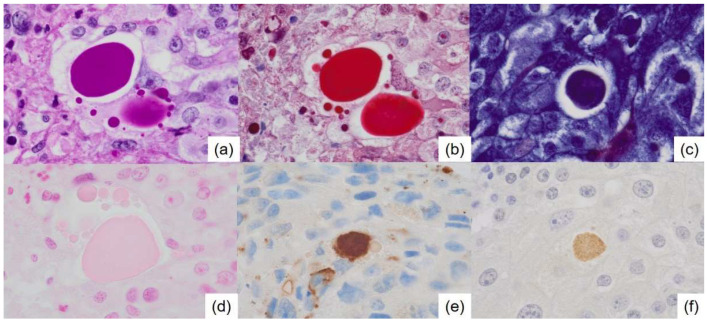
Special staining and immunohistochemical staining of the thanatosomes. (**a**) Periodic acid-Schiff reaction (×1000), (**b**) Masson’s trichrome stain (×1000), (**c**) Mallory’s PTAH stain (×1000), and (**d**) Alcian blue (×1000). The thanatosomes are positive for cleaved caspase-3 (**e**) (×1000) and ubiquitin (**f**) (×1000).

**Figure 6:**
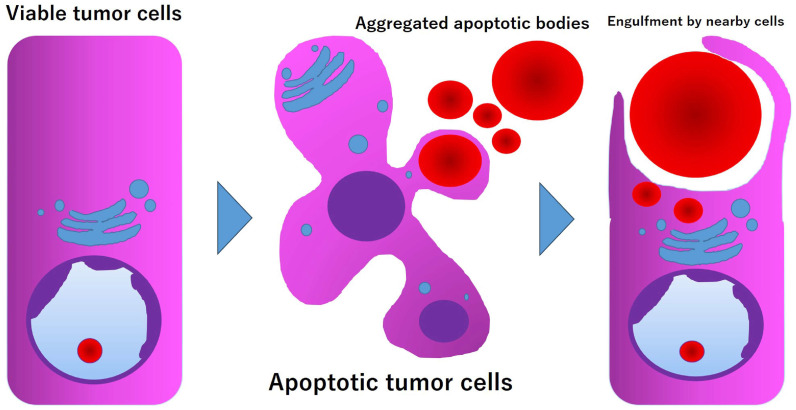
A model for forming hyaline globules in the current neoplasm. An intact tumor cell responds to various injurious stimuli, ultimately leading to its demise through a process known as programmed cell death, also called apoptosis. The apoptotic bodies are engulfed by neighboring healthy cells.

## Data Availability

Not applicable.

## Abbreviations

The following abbreviations are used in this manuscript:

HG	Hyaline globule
HER2	Human epidermal growth factor receptor type 2
